# Coagulation functions and factors correlated with central nervous system infection in herpes zoster patients

**DOI:** 10.3389/fimmu.2025.1511901

**Published:** 2025-05-09

**Authors:** Yanrong Yuan, Jing Gu, Huili Liu, Yu Wang, Zeyu Sun, Dongli Pan, Yongxing Yan

**Affiliations:** ^1^ Department of Neurology, Hangzhou Third People’s Hospital, Hangzhou, Zhejiang, China; ^2^ State key Laboratory for Diagnosis and Treatment of Infectious Diseases, National Clinical Research Center for Infectious Diseases, Collaborative Innovation Center for Diagnosis and Treatment of Infectious Disease, The First Affiliated Hospital, Zhejiang University School of Medicine, Hangzhou, Zhejiang, China; ^3^ Department of Medical Microbiology and Parasitology, Zhejiang University School of Medicine, Hangzhou, Zhejiang, China

**Keywords:** herpes zoster, central nervous system, varicella-zoster virus, coagulation function, infection

## Abstract

**Objective:**

Reports on central nervous system (CNS) infection caused by varicella-zoster virus (VZV) reactivation are increasing, but its pathogenesis remains unclear, which causes delayed diagnosis and treatment. Some studies suggested that hypercoagulability is involved in the pathogenesis of CNS infection of VZV. This study investigated the coagulation parameters of herpes zoster (HZ) and their correlations with the VZV-related CNS infection, and provided a reference for the early diagnosis and treatment.

**Methods:**

We selected 123 consecutive patients, including 95 HZ cases and 28 VZV meningitis (VZVM) cases hospitalized due to HZ. Forty-seven patients who underwent physical examination in our hospital were used as Health controls (HCs) group. The coagulation parameters of the three groups were measured and compared, and the correlation between coagulation function parameters and CNS infection was analyzed by Logistic regression. the expression of coagulation factor in cerebrospinal fluid (CSF) proteomics of 28 VZVM patients and 11 HZ patients were analyzed.

**Results:**

Compared with HCs group, plasma Fibrinogen (Fib) and D-dimer (DD) levels in HZ and VZVM group were significantly increased (P <0.01), while there were no significant differences in other parameters (P > 0.05). There was also no significant difference in the levels of coagulation parameters between the HZ and VZVM groups (P > 0.05). Proteomic analysis of CSF revealed that there was no difference in the expression levels of Fib, Antithrombin III (AT-III), and coagulation factors VII, IX, X, XI in the HZ and VZVM patients (P > 0.05). The expression levels of coagulation factors XII and XIIIa were higher in VZVM patients than those in HZ patients (P < 0.05 and P < 0.01, respectively).

**Conclusion:**

In HZ and VZVM patients, a hypercoagulable state was observed with increased Fib and DD levels. However, hypercoagulation was not a risk factor for CNS infection, and there was no significant correlation between the elevated level and the severity of disease.

## Introduction

Herpes zoster (HZ) is a skin infectious disease caused by the reactivation of latent varicella-zoster virus (VZV) in the human body. It is characterized by vesicles and rashes of the skin in the neurodistributive area, often accompanied by pain (1). Although HZ is a self-limiting skin disease, it can cause neurological complications such as postherpetic neuralgia (PHN) and cranial neuritis. In severe cases, central nervous system (CNS) infection can occur, such as meningitis, encephalitis, myelitis, cerebellar encephalitis and other complications, and even lead to serious complications such as death, increasing the burden on families and society (2–6). However, early identification and treatment of HZ patients with CNS infection mostly achieved good prognosis. Therefore, the early diagnosis and treatment is extremely important.

However, the mechanism of VZV reactivation leading to CNS infection has not been fully clarified, which may be related to direct viral infection, sustained inflammatory response, vasculitis, hypercoagulability, etc. Some studies have also found that viremia occurs in approximately 50% of the cases of HZ in immunocompetent adults, which is believed to be an important component of the pathogenesis of CNS complications in HZ (7–9). Currently, vasculitis is postulated as the most likely pathophysiological mechanism of VZV-related CNS infection (10–12). Vasculitis can cause coagulation dysfunction, thrombosis, and vice versa. Previous studies have also found that virus infection can activate hemolytic fibrinolytic system, lead to changes in coagulation function, microthrombus, and even pulmonary embolism, disseminated intravascular coagulation (DIC) and other life-threatening complications (13–15).

Human type 3 herpesvirus VZV can cause chickenpox and shingles after infection. However, it is still unclear whether the changes in coagulation functions in HZ patients and the changes in the plasma coagulation function parameters are risk factors for VZV-related CNS infection. Although some scholars believe that hypercoagulability is involved in the pathophysiological mechanism of VZV-related CNS infections, there are few studies on coagulation function in those patients. Therefore, this study examined coagulation factors in HZ and VZVM patients to assess their correlation with CNS infection, aiming to provide initial evidence of coagulation dysfunction’s role in HZ-CNS infection and support early diagnosis and treatment.

## Materials and methods

### Subjects

A total of 123 consecutive patients with shingles who were admitted to our hospital from January 2020 to January 2023 were selected, including 28 VZV meningitis (VZVM) cases and 95 HZ cases according to clinical manifestations of shingles patients and routine cerebrospinal fluid, biochemical and VZV DNA detection, and 47 health controls (HCs) from physical examination program in our hospital. Our study has been approved by the Ethics Committee of Hangzhou Third people’s Hospital (NO.2021KA013). All procedures were conducted in accordance with the Helsinki Declaration.

Inclusion criteria: 1). Age >18; 2). All patients had skin erythema, vesicles, blisters and other clinical manifestations, which met the diagnostic criteria of HZ/VZV meningitis (16); 3). Within 2 weeks of onset; 4). Diagnosed with shingles for the first time and had not used anticoagulants in the last 2 weeks. The diagnosis of shingles is made by a dermatologist and the diagnosis of VZV meningitis is made by a neurologist.

Exclusion criteria: 1). zoster sine herpete; 2). lactating or pregnant women; 3). infections caused by pathogens other than VZV.

### Methods

#### Data collection

We collected the general information of the patients, including ages, genders, clinical characteristics (mainly including the courses of diseases, the sites of herpes, treatments, etc.), comorbidity, and previous medical records.

#### Test of plasma coagulation function

Five mL of venous blood was collected from each enrolled patient and control subject on the morning of the second day of admission by anticoagulant vessels (containing 0.2ml, 0.11mol/L sodium citrate anticoagulant) under aseptic conditions, thoroughly mixed, left at normal temperature (processed within 4 hours). Plasma was collected by centrifugation at 3000r/min for 15 minutes. Levels of fibrinogen (Fib) and D-dimer (DD), prothrombin time PT), active partial thromboplastin time (APTT), thrombin time (TT) were measured on the automatic coagulation analyzer CS-5100 (Sysmex Corporation, Japan) in accordance with the operating procedures.

#### Routine and biochemical tests of cerebrospinal fluid

According to the clinical manifestations, lumbar puncture was performed within 48 hours after admission for patients with suspected VZV-related CNS infection with the consent of the patients or their families, and 6 mL cerebrospinal fluid was obtained with sterile test tubes. White blood cell count, glucose, protein, chlorine, adenosine deaminase (ADA), lactate dehydrogenase (LDH) and VZV DNA concentrations were determined.

#### Cerebrospinal fluid proteomics

CSF level of 8 coagulation proteins (Fibrinogen, Antithrombin-III, Coagulation factor VII, IX, X, XI, XII, XIIIa) were extracted from previously collected CSF proteomics dataset of the same 28 VZV meningitis and 11 herpes zoster (HZ) patients (17). Briefly, the proteins from CSF samples analyzed by mass spectrometry using data-independent acquisition (DIA) method for quantitation. Relative protein intensity in each sample was log2 transformed prior to statistical analysis.

#### Statistical analysis

SPSS 20.0 software (Chicago, IL, USA) was used for data processing and statistical analysis. Normal distribution data of measurement data were expressed as mean ± standard deviation (x ± SD), and non-normal distribution data were expressed as M (Q1 ~ Q3). One-way analysis of variance (ANOVA) was used to compare the mean of multiple groups; S-N-K method was used to test the comparison between two groups; counting data were expressed by frequency and percentage; Chi-square test or Fisher exact test was used for comparison between groups. Univariate and multivariate logistic regression analysis were performed to identify independent risk factors, and Pearson analysis was used for correlation analysis. P< 0.05 (two-tailed) was considered statistically significant.

## Results

### Baseline characteristics of patients

According to the inclusion/exclusion criteria, 95 HZ patients were included: 45 males and 50 females, with ages 23 to 88 years (59.6 ± 14.5 years). There were 28 patients with VZVM: 21 males and 7 females, with ages from 24 to 88 years (56.5 ± 16.3). The HCs group consisted of 47 cases: 26 males and 21 females, with ages from 24 to 88 years (62.2 ± 16.1 years). There was no significant difference (P>0.05) in age and comorbidity (hypertension, diabetes, stroke, coronary heart disease, tumor, chronic obstructive pulmonary disease, immune diseases, immune drug use, etc.) among the three groups except gender (**Table 1**).

**Table 1:** Baseline characteristics of patients in different groups on admission.

Characteristics	VZVM group (n=28)	HZ group (n=95)	HCs group (n=47)	F/x^2^	P
Age (mean ± SD) (years)	56.5 ± 16.3	59.6 ± 14.5	62.2 ± 16.1	1.256	0.2875
Gender					
Male (numbers,%)	21 (75.0%)**	45 (47.4%) 50 (52.6%)	26 (55.3%)	6.6875	0.0353
Female (numbers,%)	7 (25.0%)		21 (44.7%)		
Course of disease (days)	6.1 ± 4.4	5.5 ± 3.5	NA	0.6693	0.5046
Comorbidity (cases)					
Coronary heart disease	2	9	7	1.396	0.4976
COPD	0	4	2	1.2266	0.5416
Hypertension	10	35	23	2.1729	0.3374
Immune diseases	1	4	3	0.4271	0.8077
Use of immune drugs	0	3	2	1.1484	0.5631
Stroke	1	5	6	3.1688	0.2051
Diabetes	3	13	9	1.1744	0.5559
Chronic kidney disease	0	7	1	3.5812	0.1669
Chronic liver disease	1	2	1	0.2167	0.8973
Tumor	0	6	1	2.8365	0.2421
Herpes zoster site (numbers)					
Head and face	20	59	NA	0.8182	0.3657
Neck	3	5	NA	1.0567	0.304
Chest and back	4	18	NA	0.32	0.5716
Waist	1	12	NA	1.8781	0.1705
Limb	1	7	NA	0.5127	0.474

**P<0.01 *vs* HZ group; NA, not applicable; COPD, chronic obstructive pulmonary disease.

In the HZ group, shingle were found in 59 cases located on the head and face, 5 on the neck, 18 on the chest and back, 12 on the waist and 7 on the limbs. Among the 28 VZVM patients, 20 on the head and face, 3 on the neck, 4 on the chest and back, 1 on the waist and 1 on the limbs. There was no difference in shingle sites between the two groups (P>0.05). The proportion of males in VZVM group was significantly higher than the HZ group (P<0.01).

### Comparison of coagulation parameters among three groups

**Figure 1:**
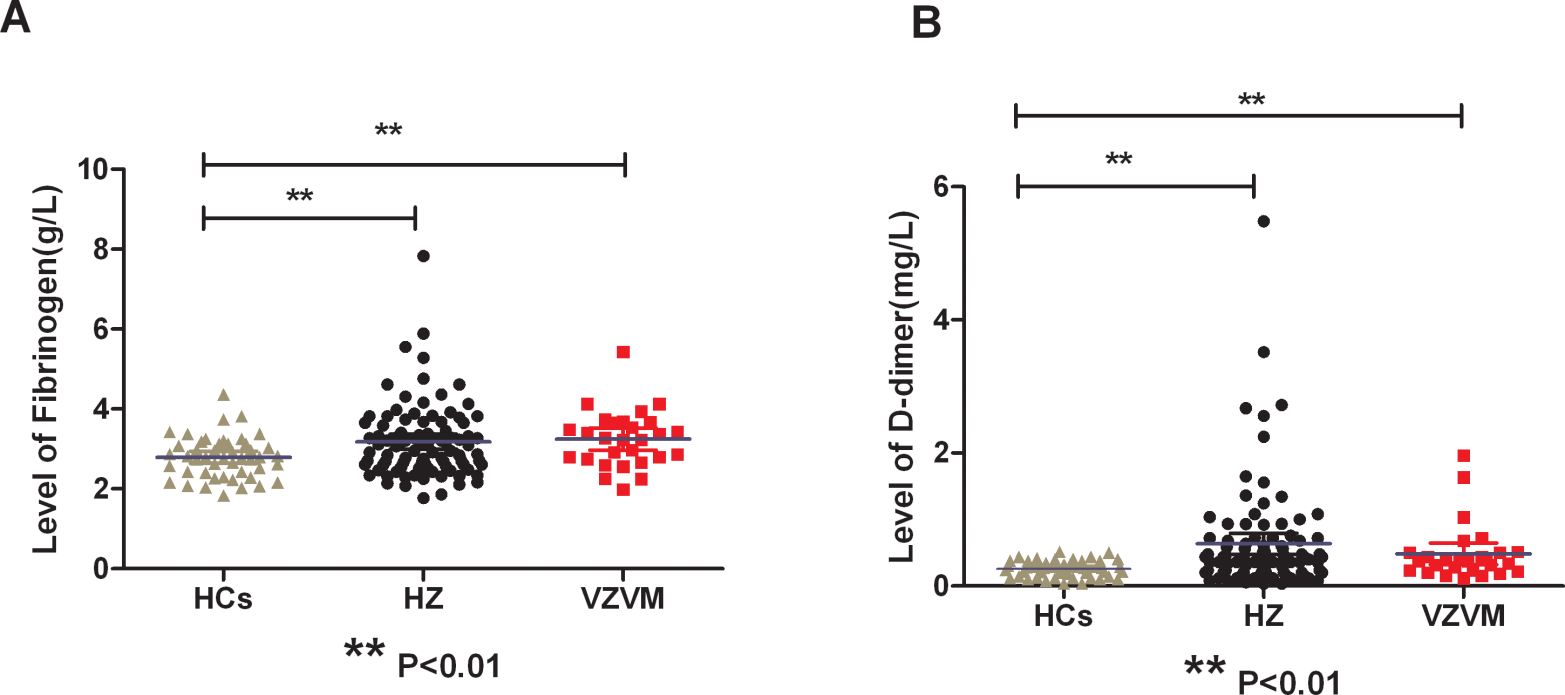
Comparison of plasma Fib **(A)** and DD **(B)** levels among three groups. HCs, health controls; HZ, herpes zoster; VZVM, varicella-zoster virus meningitis. ** P<0.01.

There were significantly increased levels of circulating Fib and DD in HZ and VZVM patients compared to the HCs (F=4.627, P<0.01; F=5.791, P<0.01), but there were no significant differences in PT, APTT, INR and TT levels among the three groups (P>0.05), as shown in **Figure 1**, **Table 2**. Inter-group comparison showed that there was no significant difference in coagulation parameters between the HZ group and the VZVM group (P>0.05).

**Table 2:** Comparison of coagulation parameters among three groups.

Characteristics	VZVM (n=28)	HZ (n=95)	HCs (n=47)	F	P
PT(sec)	11.2 ± 0.7	11.3 ± 0.7	11.4 ± 0.7	0.7124	0.4920
INR	1.0 ± 0.1	1.0 ± 0.1	1.0 ± 0.1	0.6024	0.5487
Fib(g/L)	3.2 ± 0.7**	3.2 ± 0.9**	2.8 ± 0.5	4.6270	0.0111
TT (sec)	19.0 ± 1.0	18.9 ± 1.2	18.7 ± 1.5	0.4263	0.6536
APTT (sec)	27.0 ± 1.7	26.7 ± 2.4	26.6 ± 2.0	6.3924	0.6761
DD (mg/L)	0.4 (0.2,0.5)**	0.4 (0.2,0.7)**	0.2 (0.1,0.4)	5.7910	0.0037

**P<0.01 *vs* HCs group. PT, prothrombin time; INR, international normalized ratio; Fib, fibrinogen; TT, thrombin time; APTT, active partial thromboplastin time; DD, D-dimer.

### Multivariate logistic regression analysis

Multivariate logistic regression analysis was performed with the occurrence of meningitis as the dependent variable, and the factors with P<0.5 in univariate analysis (age, course of disease, gender, Fib, DD) as the independent variables (**Table 3**). Male was the independent risk factor for VZV meningitis in HZ patients. However, coagulation function parameters and other factors were not independent risk factors (P>0.05).

**Table 3:** Multivariate logistic regression analysis of meningitis in HZ patients.

Variable	β value	SE value	Wald	OR value	95%CI	P value
Age	-0.008	0.015	0.298	0.992	0.962-1.022	0.585
Course of disease	0.067	0.056	1.425	1.070	0.958-1.194	0.233
Gender	-1.359	0.526	6.684	0.257	0.092-0.720	0.010
Fib	-0.056	0.107	0.277	0.945	0.767-1.166	0.598
DD	-0.359	0.427	0.706	0.698	0.302-1.614	0.401

Fib, fibrinogen; DD, D-dimer.

### Correlation analysis of plasma coagulation function and CSF parameters in VZVM patients

All 28 VZVM patients and 11 of the 95 HZ patients completed lumbar puncture and CSF examination. In patients with VZVM group, the white blood cell counts and protein content in CSF were all higher than the normal range, but the levels of glucose, chlorine, ADA and LDH in CSF were in the normal range. There was no significant correlation between blood coagulation parameters and the levels of white blood cell counts, protein and glucose in CSF (P>0.05).

### Comparison of coagulation factors in CSF proteomes in 28 VZVM patients and 11 HZ patients

**Figure 2:**
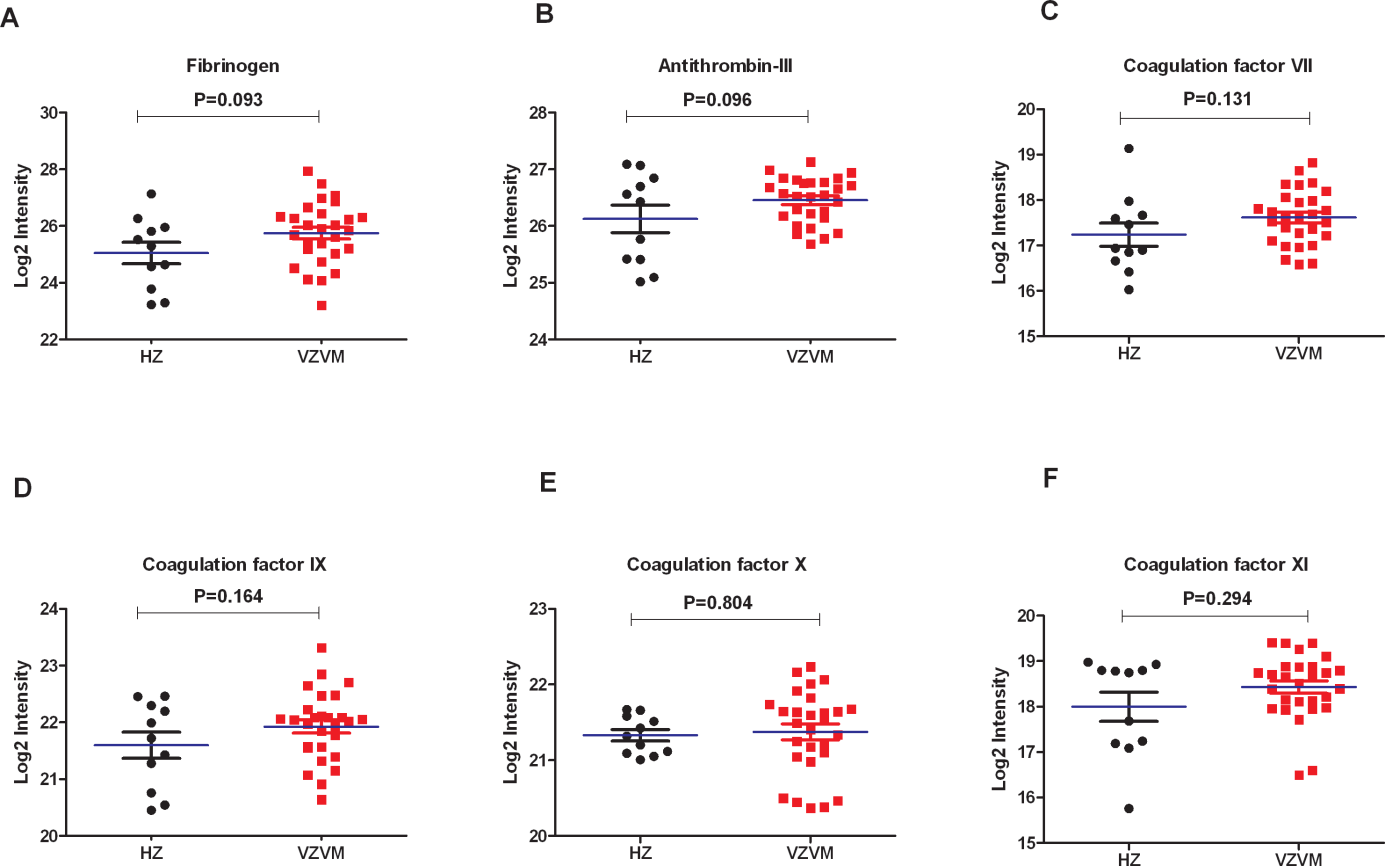
The expression levels of Fib **(A)**, antithrombin III **(B)** and coagulation factor VII **(C)**, IX **(D)**, X **(E)**, XI **(F)** in CSF between HZ and VZVM groups.

**Figure 3:**
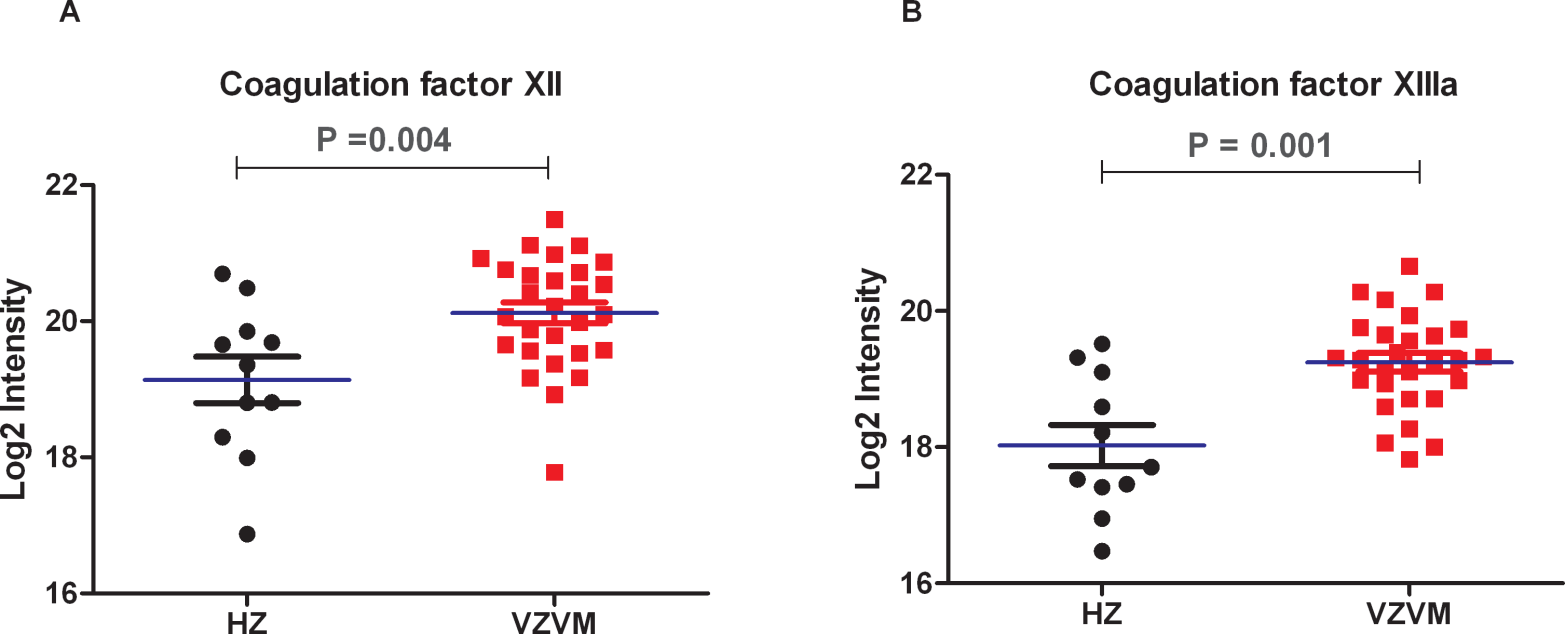
The expression levels of coagulation factors XII **(A)** and XIIIa **(B)** in VZVM group and HZ group.

Previously reported CSF proteomics data for 28 VZVM and 11 HZ patients (17) was reinterpreted to focus on coagulation markers. Compared with HZ patients, there were no differences in the expression levels of Fib, AT-III and coagulation factor VII, IX, X, XI in VZVM patients (P>0.05, **Figure 2**), but the expression levels of coagulation factor XII and XIIIa in VZVM patients were significantly higher than those in HZ patients (P<0.05, P<0.01, **Figure 3**).

## Discussion

Herpes zoster is caused by reactivated VZV, mostly in middle-aged and elderly people. With global rising of HZ incidence, 20% to 30% of people suffer from HZ in their lifetime (18, 19), and 50% of people with HZ will have a concomitant VZV viremia (7, 8). Moreover, the patients may develop various complications upon onset, including lethal CNS complications. In the case of HZ, early and timely treatment can significantly reduce the incidence of complications. Older age, diabetes mellitus, immunosuppression, and treatment delay might serve as risk factors for CNS infection in HZ patients (10, 11, 20). Kim et al. (21) found that the incidence of VZV-related meningitis in male was significantly higher than that of female. In agreement, male gender was independently associated with an increased risk of meningitis in our study.

Viral invasion can trigger homeostatic disorders within the host, including changes in coagulation function, and even lead to severe complications such as pulmonary embolism and life-threatening DIC (13–15). The parameters of coagulation function include APTT, PT, TT, Fib and DD, etc. Monitoring coagulation biomarkers which correlate with disease severity can inform therapeutic strategies. Limb et al. (22) first reported a case of amputation in an adult male with shingles complicated by pneumonia and peripheral venous thrombosis in 2009. Since then, deep vein thrombosis has been confirmed repeatedly in HZ patients (23–25), while pulmonary embolism can also be complicated in shingles patients (26–28), and the risk of stroke is significantly increased (29, 30). These results indicate that VZV infection can also lead to changes in blood coagulation function in patients. In this study, we found plasma Fib and DD levels were significantly increased in patients with HZ and meningitis as compared to HCs. As a liver-derived coagulation protein, fibrinogen (Fib) primary substrate for both hemostasis and thrombosis, and serves as a principal determinant of coagulation capacity (31, 32). In addition, Fib also binds to platelet membrane glycoprotein IIb/IIIa to mediate platelet aggregation, thus affecting blood viscosity. Increasing Fib level will lead to increased fibrin leveland therefore creats a hypercoagulable state (33). DD is one of the degradation products of fibrin and fibrinogen after activation of coagulation-fibrinolytic system, and is a marker reflecting fibrinolytic activity *in vivo*. The increase of plasma DD often occurs in patients with venous thrombosis, infection, hypercoagulable state, etc. (34, 35). This study reveals that acute herpes zoster infection is characterized by enhanced coagulation functions, compensatory fibrinolysis activation, and consequent disruption of hemostatic balance, resulting in a prothrombotic state. However, our study found that although the plasma Fib and DD levels in patients with VZV meningitis were higher than those in the control group, there was no significant difference in all coagulation function parameters compared with HZ patients. Further analysis showed that abnormal coagulation function was not an independent risk factor for meningitis in patients with shingles. Therefore, we speculate that hypercoagulability is not the pathophysiological mechanism of VZV meningitis, which needed to be validated in further studies.

CSF composition changes resulting from microbial or macromolecular infiltration correlate with CNS microenvironment alterations, serving as potential lesion biomarkers. Proteomics provides comprehensive protein quantification in biological systems, aiding pathophysiological understanding. Proteomic analyses of brain or CSF in normal and diseased states, helps to identify disease-associated proteins with diagnostic, therapeutic and prognostic relevance (36–38). Our previous study (17) on CSF proteomics in patients with VZV meningitis also found that the expression of CXCL10, IL-1RN, MPO, PRTN3 and other proteins which reflecting inflammation and immune cell activation were significantly increased. Ramachandran et al. have also found inflammatory markers elevated in the CSF during VZV-related meningitis, including interferon gamma, interleukins IL-6, IL-8, IL-10, IL-17F, IL-1RA, chemokines CXCL-9, CXCL-10, CCL-2 and G-CSF (39). Such inflammatory response is closely related to the coagulation system, and there is mutual regulation between the two systems (40). The inflammatory response of the brain can lead to increased clotting function, and anticoagulation can reduce the damage of the inflammatory response (41, 42). It is likely that the observed upregulation of coagulation factors XII and XIIIa in the CSF of VZV meningitis patients is more consistent with an inflammatory response causing BBB permeability rather than with intrinsic coagulation pathway activation. Therefore, based on the results of this study, we speculate that hypercoagulable state is not involved in the pathogenesis of VZV meningitis. The pathogenesis of VZV meningitis may be different from that of VZV encephalitis, and further comparative studies are needed to clarify it in the future. A retrospective study by Kim et al. to identify risk factors for CNS infection in 578 acute HZ patients, including 24 cases of HZ-related meningitis, in addition to discovering that males are a risk factor for VZVM, they also found that meningitis were more common in patients who had shingles in the craniocervical dermatomes, skin lesions on craniocervical distribution is also a risk factor for subsequent meningitis, and they pointed out that hematogenous invasion of virus may play a role in CNS VZV infections (21). According to studies by Satyaprakash and Kennedy et al. (7, 8), approximately 50% of herpes zoster patients have viremia, which is an important pathophysiological mechanism for the concurrent CNS infection in HZ patients. Is the inflammatory response in VZVM patients caused by viremia? It is possible that the more severe cases of HZ with meningitis are among those 50% of HZ patients who have a viremia. In future studies, we will consider testing for viremia by measuring VZV DNA in the blood of HZ and VZVM patients by PCR technology.

A limitation of this study is the relatively small cohort of VZV meningitis patients, particularly the limited number of HZ cases who underwent diagnostic lumbar puncture for CSF analysis, which may have introduced bias in our findings. Moreover, the absence of longitudinal data tracking individual patients’ pre- and post-treatment progression limits our ability to establish prognostic indicators for clinical outcomes. In the future, larger scale and multi-center studies are needed to further explore the effects of coagulation screening indicators on clinical outcomes and the need for preventive antithrombotic therapy.

## Conclusions

Reactivation of VZV can lead to changes in coagulation function in the host. Both HZ and VZV meningitis patients have hypercoagulable internal environment, mainly characterized by elevated fibrinogen and D-dimer, but such environment does not seem to be a risk factor for CNS infection. Disruption of the BBB in patients with VZV meningitis may lead to changes in partial coagulation parameters, but the pathogenesis of CNS infection in HZ may not be related to hypercoagulability. Further large-scale, multi-center, controlled studies are needed to clarify it in the future, and simultaneously detect viremia to verify our hypothesis.

## Data Availability

The raw data supporting the conclusions of this article will be made available by the authors, without undue reservation.
